# Preparation and Characterization of Bacterial Cellulose–Polyvinyl Alcohol Composite Hydrogels Using ZnCl_2_ Hydrates as Solvent

**DOI:** 10.3390/gels12030203

**Published:** 2026-02-28

**Authors:** Woradej Pichaiaukrit, Theerapat Chanamuangkon, Sujin Chumprasert, Pannagorn Sae-ear, Pichit Boonkrong, Anuchan Panaksri, Nuttapol Tanadchangsaeng

**Affiliations:** 1 College of Dental Medicine, Rangsit University, 52/347 Muang-Ake, Phaholyothin Road, Lak-Hok, Muang, Pathumthani 12000, Thailand; 2 Biomaterial Testing Center, Faculty of Dentistry, Chulalongkorn University, 34 Henri-Dunant Road, Wangmai, Patumwan, Bangkok 10330, Thailand; theerapat.ch@chula.ac.th; 3 Oral Biology Research Center, Faculty of Dentistry, Chulalongkorn University, 34 Henri-Dunant Road, Wangmai, Patumwan, Bangkok 10330, Thailand; sujin.c@chula.ac.th (S.C.); pannagorn.s@chula.ac.th (P.S.-e.); 4 College of Biomedical Engineering, Rangsit University, 52/347 Muang-Ake, Phaholyothin Road, Lak-Hok, Pathumthani 12000, Thailand; pichit.bk@rsu.ac.th (P.B.); anuchan.p@rsu.ac.th (A.P.); nuttapol.t@rsu.ac.th (N.T.)

**Keywords:** bacterial cellulose, polyvinyl alcohol, zinc chloride hydrate, composite hydrogel

## Abstract

Bacterial cellulose (BC) is highly valued for biomedical and industrial applications due to its exceptional biocompatibility, strength, and biodegradability. Polyvinyl alcohol (PVA) exhibits favorable characteristics, making it an ideal candidate for hydrogel formulation. In this study, BC–PVA composite hydrogels were synthesized by dissolving 1% *w*/*w* BC in ZnCl_2_ 3H_2_O and 10% *w*/*w* PVA in ZnCl_2_ *n*H_2_O, *n* = 6, 9, 12, and 15. These solutions were combined at BC:PVA weight ratios of 3:1, 1:1, and 1:3, then crosslinking using a glutaraldehyde–acetone solution before immersion in deionized water. The resulting hydrogels exhibited a dense, tightly packed structure with mild to moderate porosity. FTIR analysis confirmed molecular interactions via a broad, reduced O–H stretching band and the appearance of C-H bending vibrations. The water content and swelling ratio ranged from 88.13% to 94.67% and 437.93% to 997.22%, respectively. At a compressive strain of 30%, the compressive strength ranged from 62.28 kPa to 93.16 kPa. This work introduces a novel and efficient method for preparing BC-PVA hydrogels using ZnCl_2_ hydrate solvents. Both the ZnCl_2_ hydration level and the BC:PVA ratio significantly influenced the structural, water content, swelling, and mechanical properties, offering tunable materials for biomedical or industrial applications.

## 1. Introduction

Bacterial cellulose (BC) is a highly pure form of cellulose composed of linear chains of β-D-glucopyranose units linked via β-1,4-glycosidic bonds, resulting in a high density of surface hydroxyl groups. BC is biosynthesized by several Gram-negative bacteria species, most notably those within the genera *Komagataeibacter*, *Acetobacter*, *Agrobacterium*, *Pseudomonas*, and *Sarcina* [1]. Among these, *Komagataeibacter xylinus* is recognized for its superior production yields. Structurally, BC consists of a three-dimensional network of interwoven microfibrils stabilized by extensive intra- and intermolecular hydrogen bonding. Due to its exceptional physicochemical properties—including high crystallinity, thermal stability, mechanical strength, and biocompatibility—BC has attracted significant interest for diverse industrial and biomedical applications [2].

Despite these advantages, the commercial utility of BC is often hindered by its insolubility in water and common organic solvents, a characteristic rooted in its high degree of crystallinity (often >80%) and a dense, complex network of inter- and intramolecular hydrogen bonds. The equatorial hydroxyl groups on the glucopyranose rings facilitate strong cohesive forces between adjacent cellulose chains, forming robust microfibrils that resist solvent penetration.

From a practical standpoint, this recalcitrance necessitates the use of specialized, often harsh, solvent systems or energy-intensive processes for dissolution and regeneration. This lack of solubility limits the versatility of BC in forming composite materials, as it prevents molecular-level blending with other functional polymers in liquid phases. Consequently, many conventional BC-based composites suffer from weak interfacial bonding or non-homogeneous distribution, thereby restricting their application in high-performance biomedical devices.

However, BC can be dissolved in specific non-derivatizing solvent systems that disrupt its hydrogen-bonding network [3]. This dissolution occurs either through hydrogen bonding between solvent components and BC hydroxyl groups or via coordination bonding between metal ions [3]. Effective solvents include ionic liquids, amine oxides, aqueous alkali solutions, and inorganic molten salt hydrates [3].

Inorganic molten salt hydrates feature a water-to-salt molar ratio approximating the coordination number of the most strongly hydrated cation, with water molecules tightly bound within the inner coordination sphere [3]. These solvents are advantageous due to their cost-effectiveness, ease of preparation, environmental safety, and recyclability. Notable examples include LiClO_4_ 3H_2_O, LiI 2H_2_O, LiSCN 2H_2_O, and ZnCl_2_ 3H_2_O [3]. Zinc chloride hydrate, ZnCl_2_ *n*H_2_O, is a potent solvent for BC, with its efficacy being highly dependent on the hydration state (*n*) [4]. Lu and Shen (2011) demonstrated that 5.5 wt% BC could be dissolved in ZnCl_2_ 3H_2_O at 80 °C within two hours [5].

Polyvinyl alcohol (PVA) is a synthetic, water-soluble polymer valued for its excellent film-forming ability, high tensile strength, and biocompatibility. In the packaging industry, PVA is utilized as a water-soluble barrier film [6,7]; in textiles, it serves as a sizing agent; and in biomedicine, it is widely employed for wound dressings [8], drug delivery systems [9,10], and tissue engineering.

Current strategies for fabricating BC-PVA composites generally follow in situ or ex situ pathways. In the in situ approach, PVA is introduced into the culture medium during biosynthesis [11,12]. In the ex situ method, preformed BC membranes are typically immersed in PVA solution, followed by chemical crosslinking or physical processing (e.g., freeze-thaw cycles) [11,13]. To date, however, the preparation of BC-PVA hydrogels via the simultaneous dissolution of both polymers in zinc chloride hydrate followed by chemical crosslinking has not been reported. The comparative of fabricating approaches for BC/PVA composite hydrogels as shown in Table 1.

In this research, we strategically used the ZnCl_2_ *n*H_2_O molten salt hydrate system as a two-way reaction medium to overcome these constraints. The high capability of ZnCl_2_ molten salt hydrate to break the strong intra- and intermolecular hydrogen-bonding network of BC at moderate temperatures allowed molecular-level interpenetration with the PVA matrix, which made it preferable over other inorganic hydrates. The main innovation of this method is in the hydration-templating effect that is controlled by the salt’s hydration state. Tunable water molecules are coordinated in the Zn^2+^ coordination sphere. The molecular spacers (*n*) increase such that the environment of coordination becomes larger and more open during desorption, creating a more open and porous structure. The method gives exact control over the size and shape of the structure.

Unlike the ex situ techniques and thermal low-density structures, the method creates a unique interpenetrating network. They permit BC/PVA composites to attain high fluid absorption and mechanical strength, which are normally opposing properties in cryogelation and typical chemical crosslinking.

The objective of this study was to develop a composite hydrogel by dissolving BC in ZnCl_2_ 3H_2_O and PVA in ZnCl_2_ *n*H_2_O (*n* = 6, 9, 12, and 15), followed by crosslinking with a glutaraldehyde-acetone solution. We systematically characterized the surface morphology, chemical composition, swelling ratio, water content, and compressive strength of the resulting hydrogels. This work addresses a critical gap in the literature by examining how the hydration state of ZnCl_2_ modulates the crosslinking density, porosity, and mechanical performance of the composite, offering a tunable platform for biomedical applications.

## 2. Results and Discussion

### 2.1. Surface Morphology

**Figure 1:**
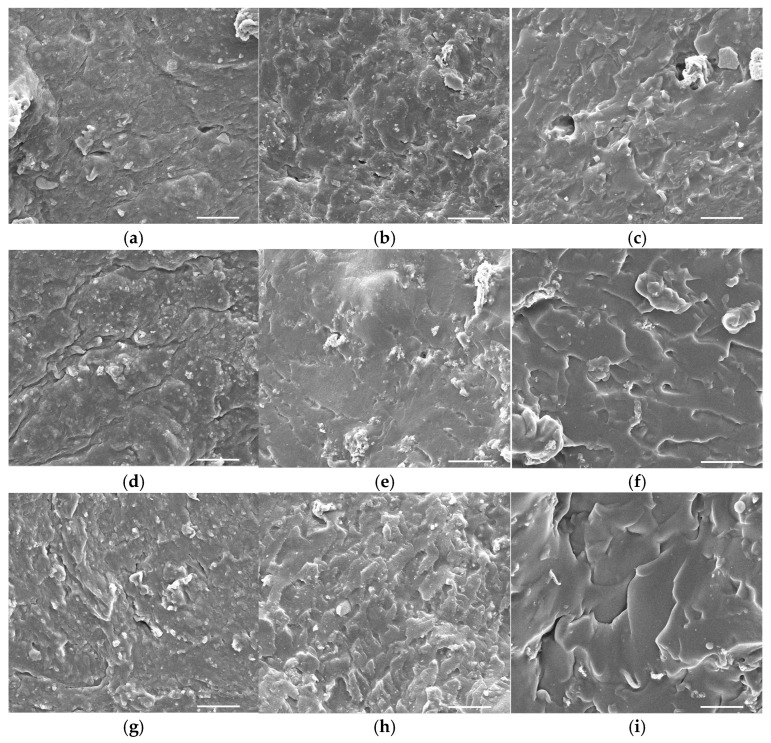

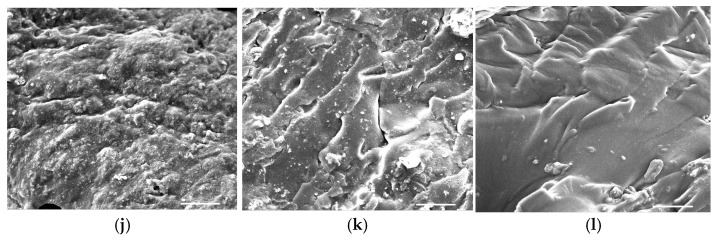
SEM micrographs depicting the surface morphology of BC–PVA composite hydrogels at 1000× magnification: (**a**) BCPVA6-I; (**b**) BCPVA6-II; (**c**) BCPVA6-III; (**d**) BCPVA9-I; (**e**) BCPVA9-II; (**f**) BCPVA9-III; (**g**) BCPVA12-I; (**h**) BCPVA12-II; (**i**) BCPVA12-III; (**j**) BCPVA15-I; (**k**) BCPVA15-II; (**l**) BCPVA15-III. The scale bar represents 50 µm.

The surface morphologies of the composite hydrogels are illustrated in Figure 1. The BCPVA6-I to BCPVA15-I groups exhibited a homogeneous, tightly packed, and dense matrix with minimal observable porosity. This compact architecture suggests a high degree of intermolecular crosslinking between the BC and PVA chains. Quantitative image analysis confirms this density, with average pore diameters measured below 10–15 µm. Conversely, the BCPVA6-II to BCPVA15-II groups displayed a dense and closely packed surface morphology characterized by moderate-to-high porosity, which is indicative of partial crosslinking. In these groups, a more defined porous network was observed, with average pore sizes generally below 10 µm, indicative of an intermediate crosslinking density.

Notably, the BCPVA6-III to BCPVA15-III groups revealed an open, fibrillar morphology defined by prominent gaps and interconnected porous channels. Quantitative measurements showed average pore diameters ranging from 10 to 20 µm. This structural configuration suggests enhanced permeability and fluid absorption capacity. Such features are particularly advantageous for biomedical applications, where facilitated cell infiltration and efficient nutrient transport are critical requirements for tissue engineering scaffolds.

### 2.2. Chemical Composition

**Figure 2:**
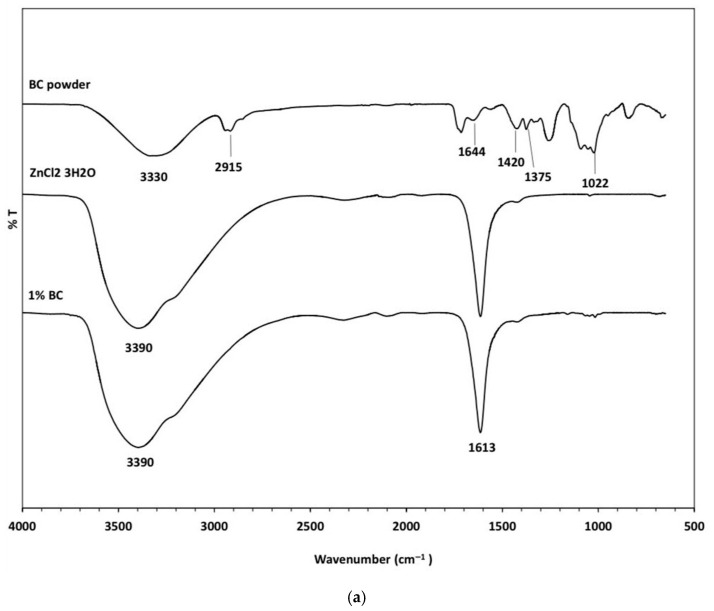

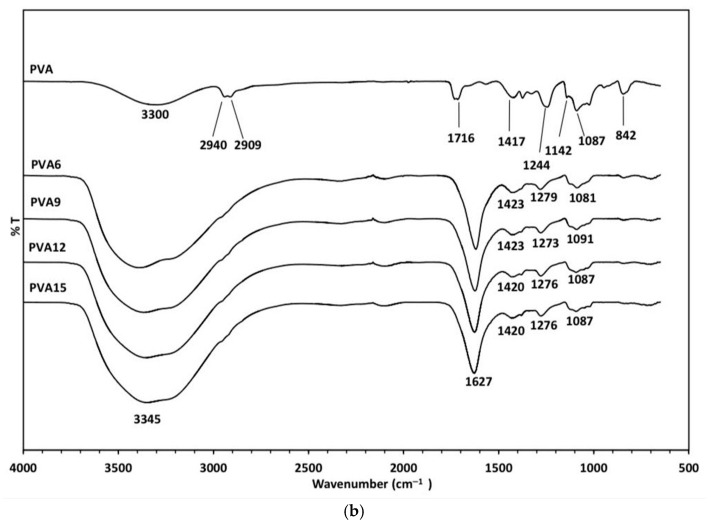
FT-IR spectra of the hydrogel components and composites: (**a**) BC powder, ZnCl_2_·3H_2_O, and 1% BC; (**b**) pure PVA and PVA6, PVA9, PVA12, and PVA15.

The FT-IR spectra of the BC powder, ZnCl_2_ 3H_2_O, and 1%BC solution are illustrated in Figure 2a. In the spectrum of the BC powder, a broad absorption band at 3300 cm^−1^ corresponds to the O-H stretching vibrations of the hydroxyl groups. As previously reported, absorption within the 3400–3440 cm^−1^ range is indicative of intermolecular hydrogen bonding in the cellulose structure, which is associated with molecular substitution [19]. The peak observed at 2915 cm^−1^ is attributed to C-H stretching vibrations, originating from the methyl/methylene groups of the cellulose backbone [19,20].

The absorption peak at 1644 cm^−1^ is assigned to H-O-H bending vibrations, confirming the presence of water molecules in the cellulose matrix, which is consistent with prior findings [19,21]. Peaks at 1420 cm^−1^ and 1375 cm^−1^ are attributed to O-H bending vibrations within the cellulose framework. Furthermore, the characteristic absorption at 1022 cm^−1^ corresponds to C-O-C stretching vibrations, representing the β-1,4-glycosidic linkages in the cellulose chains [22].

The FT-IR spectra of ZnCl_2_ 3H_2_O and the 1% BC solution reveal a broad band at 3390 cm^−1^, characteristic of O-H stretching vibrations, and a sharp peak at 1613 cm^−1^, corresponding to H-O-H bending, which confirms the presence of water molecules [23]. Notably, the characteristic peaks at 1420 and 1375 cm^−1^ were absent; this is likely due to the coordination between the cellulose hydroxyl group and the hydrated zinc cations of ZnCl_2_·3H_2_O [4].

Furthermore, the signal for the glycosidic linkage (1022 cm^−1^) was not observed, suggesting a degree of degradation under the acidic conditions inherent to the Zn^2+^ environment [4]. The H-O-H bending vibration observed in the 1% BC sample is attributed to the direct coordination of BC hydroxyl groups in BC with hydrated zinc cations, resulting in the formation of a primary hydration shell, as proposed by Sen et al. [4].

The FT-IR spectra of pure PVA, PVA6, PVA9, PVA12, and PVA15 are presented in Figure 2b. The spectrum of pure PVA exhibits a broad absorption band in the 3200–3550 cm^−1^ range, corresponding to the O-H stretching vibrations of hydroxyl groups, which indicates the presence of both intramolecular and intermolecular hydrogen bonding [24]. Peaks observed at 2940 and 2909 cm^−1^ are attributed to the asymmetric and symmetric C-H stretching vibrations of alkyl groups, respectively [24,25].

The absorption peak at 1716 cm^−1^ is assigned to C=O stretching vibrations originating from residual acetyl groups [24]. Furthermore, the peaks at 1417 cm^−1^ corresponds to C-H bending vibrations of the CH_2_ group, while the band at 1244 and 1142 cm^−1^ are attributed to C-O stretching vibrations [24,25]. The O-H bending vibration appears at 1087 cm^−1^ [25], and the peak at 842 cm^−1^ is assigned to C-C stretching vibrations [25,26].

The FT-IR spectra of PVA6, PVA9, PVA12, and PVA15 exhibited similar characteristic peaks, including broad O-H stretching (3200–3550 cm^−1^), H-O-H bending (1627 cm^−1^), C-H bending (1420 cm^−1^), C-O stretching (1276 cm^−1^), and O-H bending (1087 cm^−1^) [24,25]. However, the intensity of the peaks at 1142 cm^−1^ and 842 cm^−1^ decreased as the molar concentrations of water in the ZnCl_2_ solvent increased. This reduction in intensity suggests potential molecular interactions or structural modifications within the PVA matrix induced by the hydration state of the ZnCl_2_ salt.

**Figure 3:**
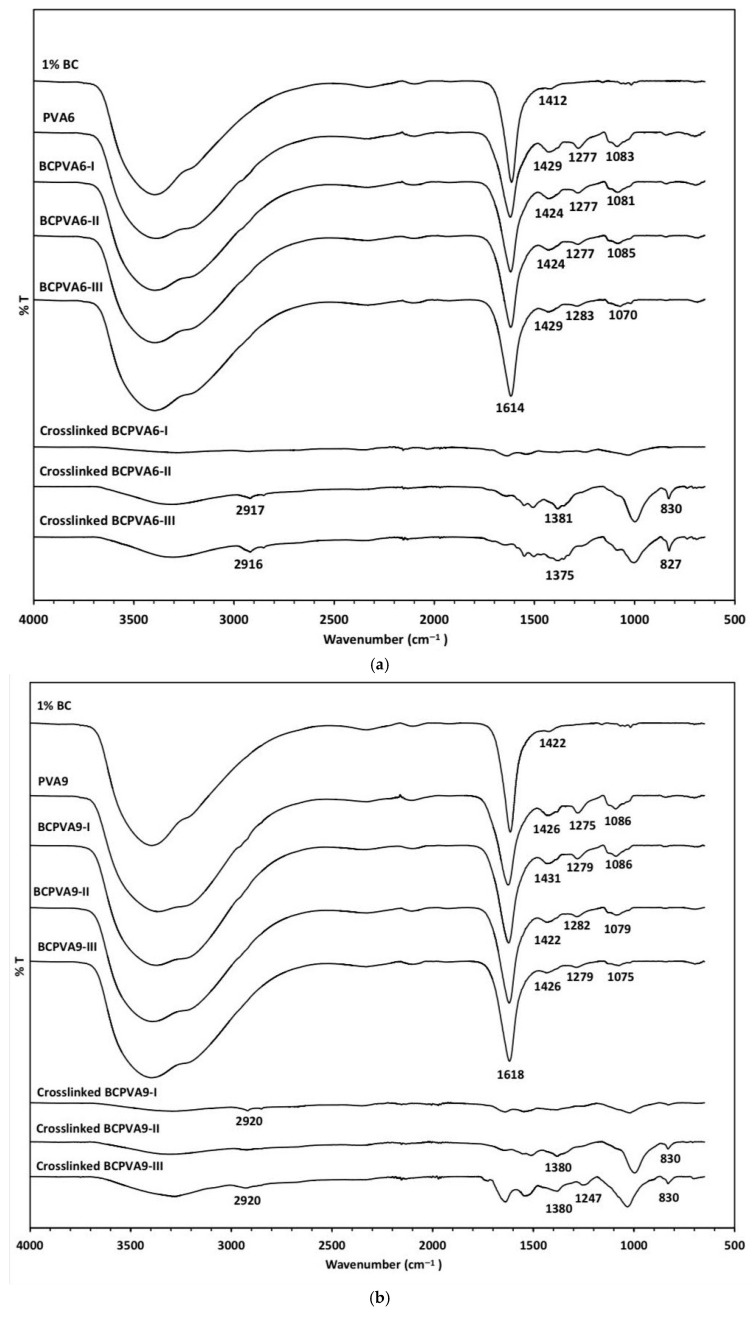

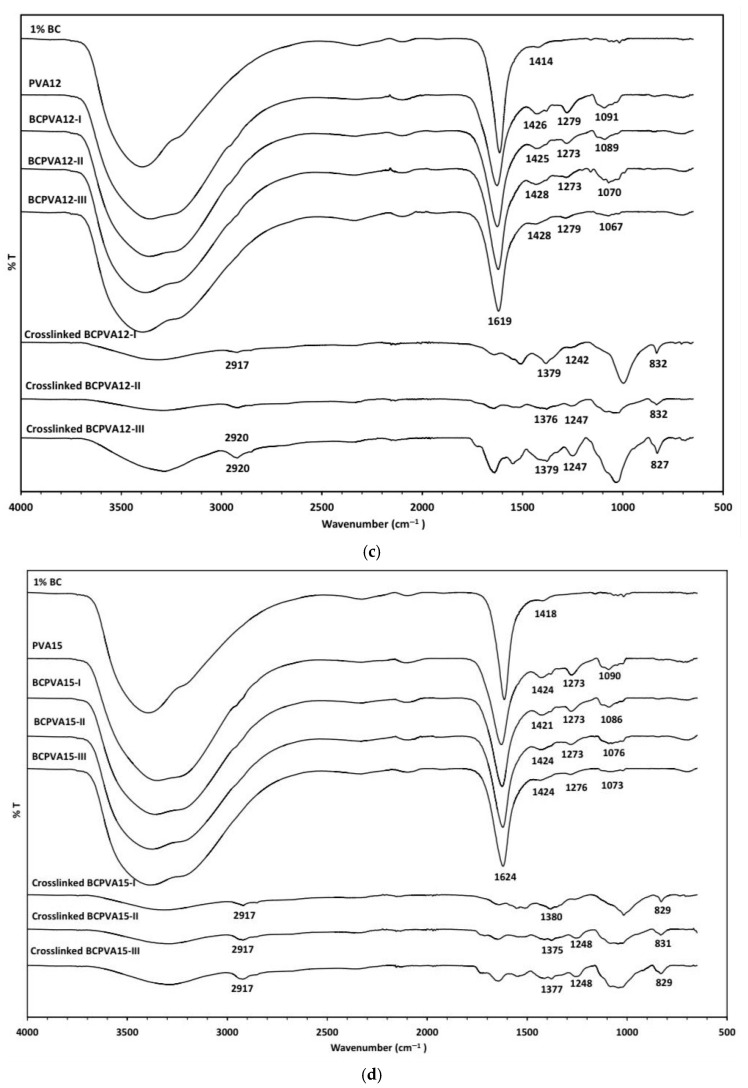
FT-IR spectra of 1% BC, pure PVA, and BCPVA composite series (I, II, and III) before and after glutaraldehyde crosslinking, categorized by ZnCl_2_ hydration levels (*n*): (**a**) *n* = 6, (**b**) *n* = 9, (**c**) *n* = 12, and (**d**) *n* = 15.

As illustrated in Figure 3a–d, the FT-IR spectra of the composite hydrogels (BCPVA6-I through BCPVA15-III) exhibited characteristic bands consistent with those observed in the corresponding PVA6, PVA9, PVA12, and PVA15. These include the broad O-H stretching band, the sharp H-O-H bending vibration, and characteristic peaks for C-H bending, C-O stretching, and O-H bending vibrations. Notably, the intensities of the C-H bending, C-O stretching, and O-H bending bands increased as the BC-to-PVA ratio decreased.

Following crosslinking, immersion in deionized water, and air drying, the FT-IR spectra (summarized in Table 2) exhibited a broad O-H stretching band with reduced intensity and the disappearance of a distinct H-O-H bending peak. Additionally, significant spectral shifts were observed: the C-H bending band shifted from approximately 1420 to 1380 cm^−1^, and the C-O stretching band shifted from around 1280 to 1250 cm^−1^. Notably, the O-H bending band near 1080 cm^−1^ vanished, while new bands attributed to C-H bending vibrations emerged in the 790–830 cm^−1^ range.

These spectroscopic transformations strictly support the proposed crosslinking mechanism driven by acid-catalyzed acetalization. The adjustment of the glutaraldehyde solution to pH 1 using HCl provided the necessary hydronium ions to activate the carbonyl groups of the glutaraldehyde, thereby facilitating a nucleophilic attack by the hydroxyl groups of the BC and PVA chains. The disappearance of the O-H bending peak at 1080 cm^−1^ and the emergence of new C-H bending vibrations (790–830 cm^−1^) serve as clear indicators of the formation of stable acetal rings. This covalent bridging effectively replaces the weaker intermolecular hydrogen bonds, creating a robust three-dimensional network. Furthermore, the significant downshift of the C-O stretching band confirms the successful transition of the polymer precursors into a permanently crosslinked composite matrix.

According to previous study [27], the crosslinking of anhydroglucose units occurs via an acetalization reaction between the carbonyl group of glutaraldehyde and the C6 hydroxyl groups of the glucose molecule. Crosslinking between PVA molecules likely proceeds through a similar mechanism involving the glutaraldehyde functional groups and the PVA hydroxyl moieties. Furthermore, the BC and PVA chains may be associated through intermolecular hydrogen bonding between the C2 and C3 hydroxyl groups of the glucose units and the hydroxyl groups of the PVA chains.

### 2.3. Water Content and Swelling Ratio

**Figure 4:**
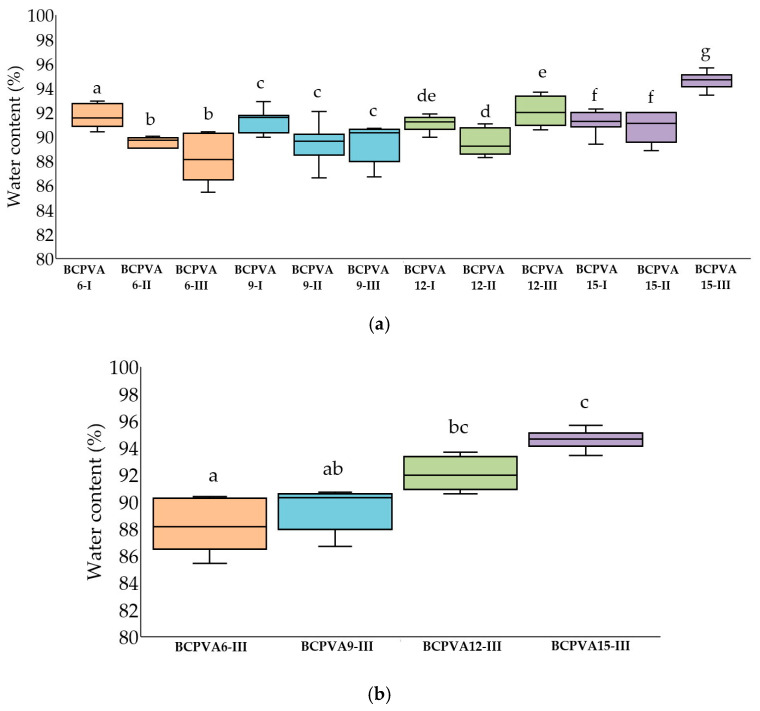
Equilibrium water content of BC/PVA crosslinked composite hydrogels. Data are presented as median values with interquartile ranges (Q1–Q3) (*n* = 7). (**a**) Intragroup comparisons within the BCPVA6, BCPVA9, BCPVA12, and BCPVA15 groups; (**b**) Intergroup comparisons among the BCPVA6-III, BCPVA9-III, BCPVA12-III, and BCPVA15-III groups. Different lowercase letters above the box plots denote statistically significant differences (*p* < 0.05) based on the Kruskal–Wallis test followed by Dunn’s post hoc analysis with Bonferroni correction. Groups sharing at least one common letter are not significantly different.

The equilibrium water content of BC/PVA composite hydrogels, synthesized with varying BC-to-PVA ratios and ZnCl_2_ hydrates of different hydration numbers, is presented in Figure 4. All samples exhibited a high water content ranging from 88.13% to 94.67%, confirming the highly hydrophilic and porous nature of the composite systems. These results are in excellent agreement with the findings of Tanpichai & Oksman (2016), who reported an average water content of approximately 92% [28].

As illustrated in Figure 4, the equilibrium water content varied significantly across the different BC/PVA hydrogels formulations. Detailed statistical parameters are summarized in Table 3 and Table 4. Statistical analysis revealed statistically significant differences within the BCPVA6 (*p* = 0.001), BCPVA12 (*p* = 0.009), and BCPVA15 (*p* = 0.001) groups. The BCPVA6 and BCPVA15 groups displayed large effect size, indicating a substantial magnitude of difference driven by the hydration state, whereas the BCPVA9 group demonstrated a comparatively lower effect size. Notably, a significant increase in water content was observed from the BCPVA6-III group to the BCPVA15-III (*p* < 0.01), with BCPVA15-III exhibiting the highest median water content.

The water retention capacity of crosslinked BC/PVA hydrogels is influenced by several synergistic factors, including crosslinking density, the BC-to-PVA mass ratio, the state of water within the matrix, and the degree of hydrogen bonding. Previous study has demonstrated that the abundant hydroxyl groups in both BC and PVA facilitate intramolecular hydrogen bonding or, competitively, establish hydrogen bonds with water molecules [29].

The hydration capacity is primarily dictated by the density of these hydrophilic functional groups and the architecture of the polymer network. The high water content observed (>88%) is consistent with the abundance of hydroxyl moieties identified via FT-IR spectroscopy. However, the variation in effect size (ε^2^) provides deeper insight into how preparation conditions influence these network properties.

In the BCPVA6 series, the high effect sizes suggests that the ratio of ZnCl_2_ 3H_2_O and ZnCl_2_ 6H_2_O significantly alters the matrix structure when BC concentration is high. Similarly, the BCPVA15 series demonstrated a large effect size, indicating that at high PVA concentrations, the hydration state of the ZnCl_2_ 15H_2_O becomes the dominant factor in determining the final water capacity. The use of ZnCl_2_ 15H_2_O introduces a larger volume of water molecules into the solvent system, which act as molecular spacers during the crosslinking process. This leads to a more expanded network with larger interstitial spaces, resulting in an open fibrillar structure that enhances water retention.

Furthermore, the mixing ratio of the polymers serves as a critical determinant of hydration behavior. In Group III, the ratio of 1% BC to 10% PVA is 1:3, the higher concentration of PVA relative to BC enhances the swelling potential and elasticity of the matrix. Conversely, the lower water content observed in Group I (3:1 ratio) may be attributed to a more rigid framework dominated by BC microfibrillar network, which restricts the volumetric expansion of the gel. These findings confirm that both the polymer composition and the solvent hydration state significantly modulate the hydrogel properties, highlighting the importance of polymer–solvent interactions in tuning the final performance of the composite.

**Figure 5:**
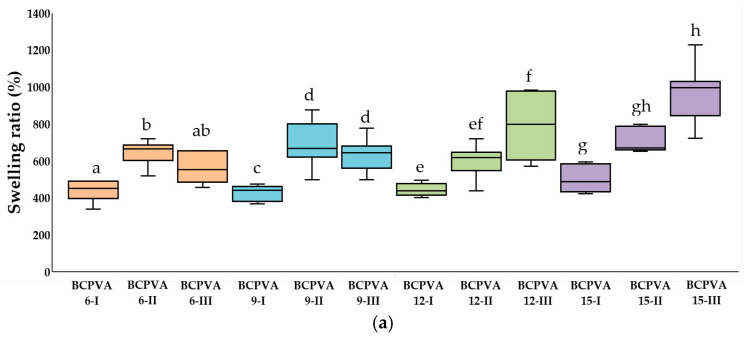

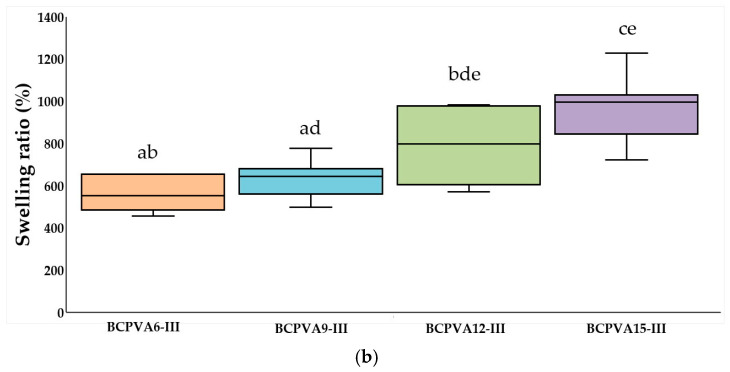
Swelling ratios of BC/PVA crosslinked composite hydrogels. Data are presented as median value with interquartile ranges (Q1–Q3) (*n* = 7). (**a**) Intragroup comparisons within the BCPVA6, BCPVA9, BCPVA12, and BCPVA15 groups; (**b**) Intergroup comparisons among the BCPVA6-III, BCPVA9-III, BCPVA12-III, and BCPVA15-III groups. Different lowercase letters above the box plots indicate statistically significant differences (*p* < 0.05) based on the Kruskal–Wallis test followed by Dunn’s post hoc analysis with Bonferroni correction. Groups sharing at least one common letter are not significantly different.

The swelling ratios of the BC/PVA hydrogels are depicted in Figure 5. The BCPV15-III group exhibited the highest median swelling ratio at 997.22%, while the BCPVA12-I group showed the lowest at 437.93%. These values exceed those reported by Tanpichai and Oksman (2016) for similar glutaraldehyde-crosslinked systems [28]. Statistical analysis (Table 5 and Table 6) revealed statistically significant differences within the BCPVA6 (*p* = 0.017), BCPVA9 (*p* = 0.001), BCPVA12 (*p* = 0.001), and BCPVA15 (*p* < 0.001) groups. The BCPVA15 grop demonstrated the largest effect size, followed by BCPVA9 and BCPVA12 groups, suggesting that these formulations are profoundly sensitive to variations in preparation conditions. Furthermore, significant differences were observed among the BCPVA6-III, BCPVA9-III, BCPVA12-III, and BCPVA15-III groups (*p* = 0.005).

Notably, the swelling ratio did not directly correlate with the initial equilibrium water content. This discrepancy is primarily attributed to the sample preparation for the swelling test; the original three-dimensional hydrogel matrix was converted into a densely packed structure with reduced porosity during the drying phase, which inherently altered the subsequent rehydration kinetics.

The transition from a hydrated state to a xerogel (dried state) involves significant structural alterations. The removal of water during air drying promotes the approximation of polymer chains, potentially leading to irreversible hornification—a phenomenon where secondary hydrogen bonding creates a more compact, less porous matrix compared to the original as-synthesized gel. However, the remarkable ability of the BCPVA15-III hydrogel to re-swell to over 997% indicates that the covalent acetal crosslinks successfully preserved the structural memory of the network. This high re-swelling capacity confirms that the hydration-templating effect creates a permanent framework capable of overcoming the physical compaction induced by drying, allowing the matrix to return to its pre-expanded state upon contact with fluids.

Consistent with the water content trends, swelling behavior was a function of the BC/PVA ratio and the hydration state of ZnCl_2_. Hydrogels with higher PVA content exhibit superior swelling ratios compare to BC-rich hydrogels. This is attributed to the high hydrophilicity of PVA and its flexible polymer chains, which undergo significant relaxation and expansion upon hydration. Conversely, BC-rich hydrogels were more resistant to swelling due to the rigid, non-expanding nature of the BC nanofibrillar framework. In the Group III series, swelling ratios increased with the hydration number of ZnCl_2_ in the following order: ZnCl_2_ 6H_2_O < ZnCl_2_ 9H_2_O < ZnCl_2_ 12H_2_O < ZnCl_2_ 15H_2_O.

The BCPVA12-III and BCPVA15-III groups exhibited exceptionally high swelling ratios exceeding 900%. This suggests that the highly hydrated ZnCl_2_ system likely reduced the effective crosslinking density during gelation, thereby enhancing polymer chain mobility and facilitating solvent diffusion into the matrix. These findings indicate that swelling behavior is governed by a synergy between network hydrophilicity, chain flexibility, and crosslink density.

The swelling capacity and water retention of the BCPVA hydrogels are fundamentally governed by the hydration-templating effect of the ZnCl_2_ *n*H_2_O system. At lower hydration states, the Zn^2+^ ions possess a smaller primary coordination sphere, resulting in a more concentrated electrolyte medium that keeps the BC and PVA chains in closer proximity during the dissolution-regeneration process. Consequently, the resulting network is denser, offering less free volume for water entrapment. As n increases to 15, the larger volume of coordinated water molecules acts as a molecular spacer. These water molecules shield the polymer chains from one another, preventing dense re-aggregation during the regeneration step. This increased inter-chain distance creates a pre-expanded framework that can accommodate significantly more fluid, explaining the leap in swelling ratio from 554.39% to 997.22%.

**Figure 6:**
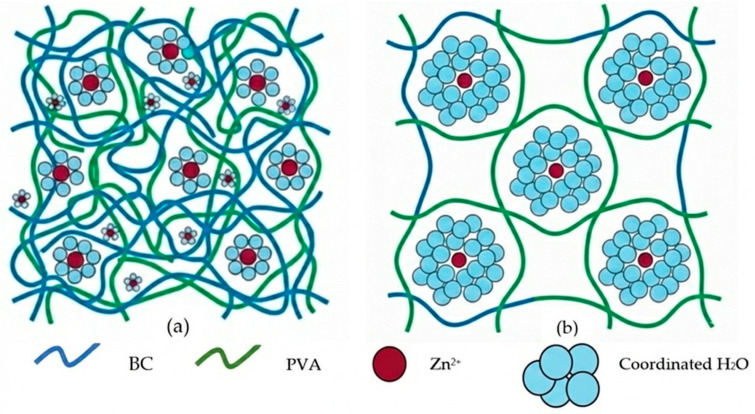
Proposed mechanistic model of the hydration-templating effect in BC/PVA hydrogels. (**a**) At a low hydration state (*n* = 6), the small coordination sphere of Zn^2+^ ions leads to close proximity of polymer chains and a dense matrix. (**b**) At a high hydration state (*n* = 15), the expanded hydration sphere acts as a molecular spacer, creating an open, porous network that enhances swelling and water retention.

The high water content (>94%) in the BCPVA15 group is also a result of the modified crosslinking density. While the acetal bonds provide structural integrity, the templated macropores, which observed in SEM, reduce the elastic contraction force of the network. This allows the osmotic pressure driven by the hydrophilic hydroxyl groups to expand the gel further than the more rigid, compact structures of the BCPVA6. The proposed mechanistic model of the hydration-templating effect in BC/PVA hydrogels as shown in Figure 6.

The achievement of swelling ratios exceeding 900% provides critical functional advantages for biomedical applications, particularly in advanced wound care and tissue engineering. In chronic wound management, hydrogels with high absorption capacity are essential for effectively sequestering heavy exudates. This prevents periwound maceration and reduces the required frequency of dressing changes, thereby improving patient comfort and healing outcomes. Moreover, the highly expanded network at equilibrium (as seen in BCPVA15-III group) ensures the presence of large, water-filled channels. These channels are vital for the efficient diffusion of oxygen, nutrients, and metabolic waste products, which is a prerequisite for supporting cell viability and proliferation within 3D scaffolds. Furthermore, the significant volumetric expansion allows these hydrogels to serve as high-capacity reservoirs for therapeutic agents. The swelling-controlled release mechanism enables the sustained delivery of drugs or growth factors, tailored to the hydration state of the environment.

The enhanced swelling and water retention capacity observed in the BCPVA-III groups can be elucidated through a hydration-templating mechanism, which differentiates this study from conventional composite fabrication. Unlike simple physical blending where polymers aggregate randomly, the use of ZnCl_2_ 15H_2_O establishes a highly organized coordination environment. Here, the bound water molecules within the Zn^2+^ inner sphere act as molecular templates that prevent the premature collapse of the BC-PVA interpenetrating network during chemical crosslinking. Upon the removal of the salt through washing, these hydrated domains transition into a well-defined, interconnected porous architecture. This specific mechanistic pathway bypasses the typical trade-off between mechanical integrity and swelling efficiency, as the expanded pores provide ample volume for water sequestration while the molecularly blended IPN maintains the structural framework.

### 2.4. Compressive Properties

**Figure 7:**
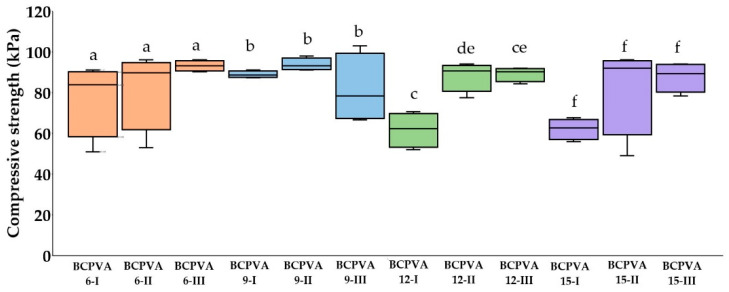
Compressive strength at 30% strain of BC/PVA crosslinked composite hydrogels. Data are presented as median value with interquartile ranges (Q1–Q3) (*n* = 4). Different lowercase letters above the box plots denote statistically significant differences (*p* < 0.05) based on the Kruskal–Wallis test followed by Dunn’s post hoc analysis with Bonferroni correction. Groups sharing at least one common letter are not significantly different.

The compressive strength of the BC/PVA composite hydrogels is presented in Figure 7, with value ranging from 62.28 kPa to 93.16 kPa. The BCPVA6-III group exhibited the highest median compressive strength, while the BCPVA12-I group showed the lowest. Significant mechanical differences were only observed within the BCPVA12 series, as detailed in the statistical analysis in Table 7 and Table 8.

In the BCPVA6 series, the compressive strength increased progressively with PVA content (BCPVA6-I < BCPVA6-II < BCPVA6-III). This suggests that a higher proportion of PVA facilitates the formation of a more stable, integrated network. This enhancement is attributed to the efficient crosslinking of PVA chains by glutaraldehyde, which increases the load-bearing capacity. The mechanical integrity is further reinforced by a complex hydrogen-bonding network consisting of intramolecular bonds within the BC fibrils and intermolecular bonds between the BC and PVA chains. In these composites, the BC nanofibrillar network acts as a structural scaffold, providing significant reinforcement to the PVA matrix.

When utilizing the ZnCl_2_ 9H_2_O system, the compressive strength reached a peak of 93.16 kPa for BCPVA9-II but decreased in the BCPVA9-III group. This reduction suggests that excessive PVA content, when combined with increased zinc hydration, may lead to steric hindrance or a dilution effect that partially disrupts effective crosslink formation during the gelation process.

In contrast, the BCPVA12-I and BCPVA15-I groups exhibited lower compressive strengths compared to their BCPVA6-I and BCPVA9-I counterparts. This indicates that a high BC proportion (BC:PVA = 3:1) combined with highly hydrated ZnCl_2_ weakens the structural integrity of the hydrogel. This increased hydration number of ZnCl_2_ likely reduces the intermolecular interactions and effective crosslink density by increasing the free volume within the matrix. However, when the BC:PVA ratio was adjusted to 1:1 and 1:3 in these highly hydrated systems, the compressive strength recovered. This suggests that the reinforcing effect of the crosslinked PVA network can compensate for the structural weakening caused by high solvent hydration.

The statistical significance observed specifically within the BCPVA12 groups (Table 7 and Table 8) highlights a critical mechanical transition point in the composite architecture. At this hydration state, the ZnCl_2_ hydration sphere provides a specific degree of molecular spacing that makes the network highly sensitive to the BC-to-PVA ratio. As shown in Figure 7, the compressive strength increases significantly from BCPVA12-I to BCPVA12-III, suggesting that at this intermediate hydration level, the increasing proportion of crosslinked PVA matrix is essential to reinforce the structural voids created by the hydration-templating effect. This sensitivity indicates a percolation threshold where the interpenetrating network transitions from a BC-dominated rigid framework to a more synergistic BC/PVA reinforced matrix.

### 2.5. Physicochemical and Mechanical Properties

**Figure 8:**
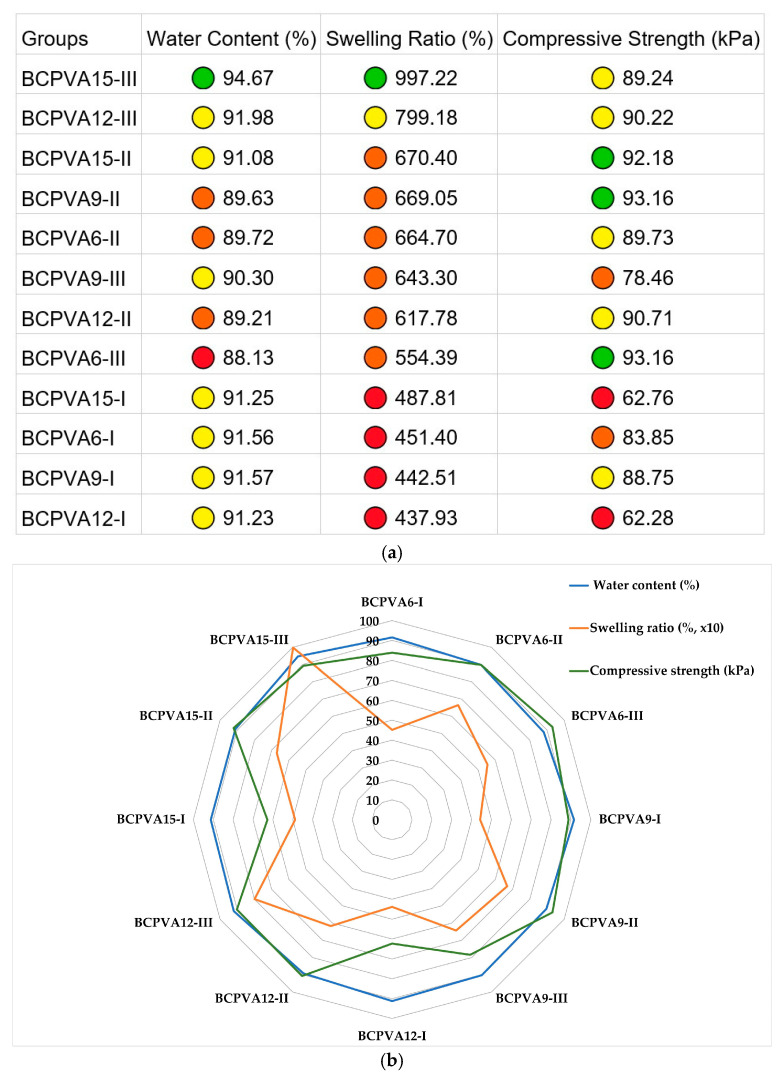
Integrated physicochemical and mechanical property profiles of BC/PVA hydrogels. Data are presented as median values. The color-coded performance clusters represent: Green (optimal performance), Yellow (high performance), Orange (moderate performance), and Red (baseline performance). (**a**) Heatmap analysis showing the distribution of water content, swelling ratio, and compressive strength across the twelve groups; (**b**) Radar chart illustrating the property trade-offs and the superior geometric area of the BCPVA15-III formulation. The swelling ratio values were scaled (×10^−1^) to allow visualization together with water content and compressive strength within a single chart.

The physicochemical and mechanical properties of the twelve BC/PVA hydrogel formulations were synthesized into a heatmap (Figure 8a) and a radar chart (Figure 8b) to identify performance clusters and structure-property correlations. The results demonstrate that most formulations maintain a high equilibrium water content (>88%) and stable compressive strength (62.28–93.16 kPa). The BCPVA15-III group emerged as the optimal candidate, occupying the largest geometric area in the radar chart. It achieved the highest median water content of 94.67% and a maximum swelling ratio of 997.22%. Statistical analysis revealed that the effect size for the swelling ratio was highest in the BCPVA15 series (ε^2^ = 0.840, *p* < 0.001), indicating that at the highest ZnCl_2_ hydration state, the swelling capacity becomes extremely sensitive to the polymer mixing ratio. Conversely, the group I formulations (BC:PVA ratio = 3:1) consistently represented the baseline zone in the heatmap, showing significantly lower swelling capacities (437.93–487.81%) compared to Groups II and III.

The integration of the radar chart and heatmap reveals that hydrogel performance is governed by the synergy between the solvent’s hydration state and the BC:PVA ratio. The use of ZnCl_2_ 15H_2_O introduces a higher volume of water molecules during the gelation process; these molecules act as molecular spacers that prevent dense chain packing. When combined with the 1:3 mixing ratio (Group III), which provides the highest density of flexible PVA chains, the network can expand to its maximum expansion potential. This is statistically supported by the strong effect sizes observed in the BCPVA15 series for both water content (ε^2^ = 0.639) and swelling ratio (ε^2^ = 0.840).

The asymmetry observed in radar chart highlights that the swelling ratio is the most structure-sensitive property. While water content remains relatively stable across formulations due to the consistent presence of hydrophilic hydroxyl groups (as confirmed by FT-IR), the swelling capacity is highly dependent on the effective crosslinking density. Interestingly, the compressive strength remained robust (89 kPa for BCPVA15-III), suggesting that the BC framework provides a consistent mechanical floor that prevents structural collapse even at near-maximal hydration levels.

For a biomedical perspective, the optimal profile is represented by the maximal area coverage of the BCPVA15-III group. Its high hydration and nearly 1000% swelling capacity are ideal for maintaining a moist wound environment and absorbing high levels of exudate. Simultaneously, the stable mechanical strength ensures that the material can withstand external pressure during clinical use. This study demonstrates that tuning the solvent’s hydration state is a highly effective strategy for engineering high-performance BC/PVA composite gels with tailored functional properties.

The results suggest that ZnCl_2_ hydration modulates hydrogel properties primarily by influencing effective crosslink density and polymer chain mobility. Higher hydration states introduce additional water molecules that act as molecular spacers during gelation, sterically hindering dense chain packing and promoting long-range network. When integrated with higher PVA content, this mechanism significantly enhances swelling capacity. Notably, the mechanical integrity is preserved through the reinforcing BC microfibrillar framework, which maintains the structural floor even as the PVA matrix undergoes maximum expansion. These findings clarify the synergetic structure-property relationships that govern the performance of BC/PVA composite systems.

## 3. Conclusions

This study successfully demonstrated the fabrication of BC/PVA composite hydrogels through a novel hydration-templating approach using a ZnCl_2_ hydrate solvent system and acid-catalyzed acetalization. Morphological and spectroscopic analyses (FT-IR) confirmed the transition from a precursor mixture to a permanently crosslinked 3D matrix, characterized by the formation of stable acetal bridges (C-O-C-O-C). The disappearance of the O-H bending peak at 1080 cm^−1^ and the emergence of new C-H vibrations (790–830 cm^−1^) serve as definitive indicators of this covalent bridging, which effectively replaces weaker intermolecular hydrogen bonds.

A key finding of this research is the high tunability of the hydrogel’s functional properties by modulating the solvent hydration state and the polymer mixing ratio. By increasing the hydration number, the Zn^2+^ coordination sphere acts as a molecular spacer, preventing dense chain re-aggregation and allowing for a pre-expanded, highly porous architecture. This mechanism directly enabled the synthesis of hydrogels with exceptionally high water content (>94%) and expansive swelling ratios exceeding 900% (specifically in the BCPVA15-III group).

Mechanically, the hydrogels maintained robust compressive strengths (62.28–93.16 kPa), with the BCPVA12 series identified as a critical percolation threshold where the synergy between the BC scaffold and the PVA matrix is most sensitive to compositional changes. Statistical analysis with high effect sizes (up to ε^2^ = 0.840) underscores the precision with which these properties can be tuned.

Given their superior balance of mechanical integrity, structural memory after drying, and high fluid absorption capacity, these BC/PVA hydrogels—particularly the BCPVA15-III formulation—hold significant potential for advanced biomedical applications. These include high-exudate wound management, where the material can effectively sequester fluids while maintaining a moist environment, and as scaffolds for tissue engineering, where the tunable macroporosity supports efficient nutrient transport and cell proliferation.

### Limitation and Future Work

While the physicochemical and mechanical results demonstrate the robust potential of BC/PVA composite hydrogels, this study primarily focuses on the hydration-templating fabrication and the resulting structure-property relationships. A primary limitation is the absence of direct biological evaluations, which are essential for clinical translation.

Future research will prioritize in vitro cytotoxicity assessments using fibroblast or keratinocyte cell lines to confirm the biocompatibility of the acetal-crosslinked network and ensure the complete removal of any residual ZnCl_2_ or glutaraldehyde. Furthermore, cell adhesion and proliferation studies are necessary to evaluate how the tunable macroporosity and high swelling capacity influence cell-material interactions and nutrient diffusion within the 3D scaffold.

Beyond in vitro testing, in vivo wound healing studies in animal models will be conducted to evaluate the hydrogel’s efficacy in exudate management and its ability to promote re-epithelialization under physiological conditions. Additionally, we recognize that rheological behavior is a critical parameter for evaluating the suitability of hydrogels in such dynamic environments; therefore, we are committed to performing comprehensive oscillatory rheology in future studies to evaluate the fatigue resistance and shear-thinning properties of these scaffolds. These biological and rheological validations will bridge the gap between the material’s current structural advantages and its practical application as an advanced biomedical dressing.

## 4. Materials and Methods

### 4.1. Materials

BC powder was kindly obtained from the National Research Council of Thailand. ZnCl_2_ was purchased from Kemaus (Cherrybrook, New South Wales, Australia). PVA (Mw = 30,000–70,000 g/mol; 87–90% hydrolyzed), glutaraldehyde (25% aqueous solution), acetone, and hydrochloric acid (HCl) were purchased from Sigma-Aldrich (St. Louis, MO, USA).

### 4.2. Preparation of BC/PVA Composite Crosslinked Hydrogel

A 1% *w*/*w* BC solution was prepared by homogenizing BC in ZnCl_2_ 3H_2_O (1% BC) at 80 °C using a hot plate equipped with a magnetic stirrer for 8 h. Separately, 10% *w*/*w* PVA solutions were prepared by dissolving PVA in ZnCl_2_ 6H_2_O (PVA6), ZnCl_2_ 9H_2_O (PVA9), ZnCl_2_ 12H_2_O (PVA12), and ZnCl_2_ 15H_2_O (PVA15) under the same conditions.

To fabricate the composite crosslinked hydrogels, BC and PVA solutions were mixed in weight ratios as shown in Table 9 and heated at 80 °C for 10 min under constant stirring to ensure homogeneity. Crosslinking was initiated by adding 3 mL of a glutaraldehyde–acetone solution to the BC/PVA blend, followed by stirring for 1 min at 80 °C. The glutaraldehyde–acetone solution was prepared by mixing 10% (*v*/*v*) glutaraldehyde (from a 25% aqueous stock) with acetone, after which the pH was adjusted to approximately 1.0 using 35% HCl, as described by Qiu et al. (2012) [15]. The crosslinked mixture was then poured into silicone molds (2.5 × 2.5 × 1 cm^3^) and left overnight at room temperature to complete the gelation process. Subsequently, the hydrogels were removed and immersed in deionized (DI) water for 48 h to remove residual crosslinking agents, with the DI water being changed periodically. The samples were stored in DI water at room temperature until further characterization.

### 4.3. Surface Morphology

The surface morphology of the hydrogels was examined using a scanning electron microscope (SEM). Hydrogel samples were cut into cubic pieces (1 × 1 × 1 cm^3^) and dried in a desiccator jar. The samples were subsequently dried using a critical point dryer and sputter-coated with gold using an evaporator coater (JFC-1200, JEOL, Tokyo, Japan). SEM imaging was performed using a JSM-IT300 microscope (JSM-IT300, JEOL, Tokyo, Japan) at an accelerating voltage of 15 kV and a magnification of 1000×.

### 4.4. Chemical Composition

Structural variations and intermolecular interactions of the hydrogel samples were analyzed using a Fourier-transform infrared (FTIR) spectrometer (Nicolet 6700, Thermo Fisher Scientific, Waltham, MA, USA) equipped with a universal attenuated total reflectance (UATR) accessory. Spectra were recorded in transmittance mode over the range from 4000 to 400 cm^−1^ using 64 scans per sample at a resolution of 8 cm^−1^. Baseline correction and spectra normalization were applied to account for variation in the sample.

### 4.5. Water Content and Swelling Ratio

Before evaluating water content and swelling ratio, the hydrogels were cut into cubic specimens with dimensions of 1 × 1 × 1 cm^3^. The samples were air-dried at room temperature until a constant weight was obtained. To determine the water content of the hydrogel, the sample was weighed before (Wi) and after drying (Wd). The water content (Wc) was calculated using the following equation:

$$Wc\;(\%)=\frac{\left(Wi-Wd\right)}{Wi}\times 100$$

To estimate the degree of swelling, dried hydrogel samples were immersed in deionized water for 120 h to reach swelling equilibrium. Before weighing, excess surface water was removed by gently blotting the swollen samples with filter paper. The equilibrium swelling ratio (S) was calculated according to the equation:

$$S\;(\%)=\frac{\left(Ws-Wd\right)}{Wd}\times 100$$

where Ws and Wd represent the weights of the swollen and dry samples, respectively.

### 4.6. Compressive Properties

The compressive properties of the hydrogels were evaluated using a QC-528M1F universal materials testing machine (Cometech Testing Machines Co., Ltd., Taichung City, Taiwan). The hydrogels were cut into cubic specimens with dimensions of 1 × 1 × 1 cm^3^. Compression tests were conducted at a strain rate of 10% min^−1^ with an applied load of 0.05 N [28]. The compressive strength was calculated at a compressive strain of 30%.

## 5. Statistical Analysis

Statistical analyses were performed using SPSS Statistics version 26 (IBM Corp., Armonk, NY, USA). The data for water content, swelling ratio, and compressive strength were analyzed for differences among groups using the Kruskal–Wallis test, with statistical significance set at *p* < 0.05.

## Figures and Tables

**Table 1:** Comparative of synthesis approaches for BC/PVA composite hydrogels.

Approach	Methodology	Structural Characteristic	Advantages	Limitations	References
In situ	Co-cultivation	Integrated fibrillar network.	Homogeneous PVA distribution within the BC pellicle.	Long fermentation; restricted polymer concentration.	[14]
Ex situ (Physical)	Impregnation	Surface-dominant coating.	Non-destructive; maintains native fiber morphology.	Weak interfacial bonding; limited penetration depth.	[14]
Ex situ (Chemical)	Covalent Crosslinking	Rigid, high-density network.	High mechanical stability; robust network integrity.	Potential cytotoxicity; reduced matrix elasticity.	[15]
Cryogelation	Freeze–thaw cycling	Macro-porous crystalline zones.	Solvent-free; high biocompatibility.	Lower swelling efficiency; requires multiple cycles.	[16]
Solvent casting	Thermal evaporation	Compact, low-void film.	Predictable thickness; high homogeneity.	High-density packing; limited fluid absorption.	[17,18]
Molten salt hydrate	Hydration-templating	High-porosity, tunable architecture.	Highly tunable porosity; superior swelling capacity (>900%); high compressive strength (~93 kPa); reduced processing time compared to in situ methods	Post-synthesis salt removal required.	This study

**Table 2:** FT-IR spectra of crosslinked and air-dried BC/PVA hydrogel.

Functional Groups	Wavenumber Range (cm^−1^)	Groups
O-H stretching (broad)	3200–3550	BCPVA6-II, BCPVA6-III BCPVA9-I, BCPVA9-II, BCPVA9-III BCPVA12-I, BCPVA12-II, BCPVA12-III BCPVA15-I, BCPVA15-II, BCPVA15-III
O-H stretching (Reduced intensity)	2700–3200	BCPVA6-II, BCPVA6-III BCPVA9-I, BCPVA9-III BCPVA12-I, BCPVA12-II, BCPVA12-III BCPVA15-I, BCPVA15-II, BCPVA15-III
C-H bending	1330–1420	BCPVA6-II, BCPVA6-III BCPVA9-II, BCPVA9-III BCPVA12-I, BCPVA12-II, BCPVA12-III BCPVA15-I, BCPVA15-II, BCPVA15-III
C-O stretching	1200–1275	BCPVA9-III BCPVA12-I, BCPVA12-II, BCPVA12-III BCPVA15-II, BCPVA15-III
C-H bending (New band)	790–830	BCPVA6- II, BCPVA6-III BCPVA9-II, BCPVA9-III BCPVA12-I, BCPVA12-II, BCPVA12-III BCPVA15-I, BCPVA15-II, BCPVA15-III

**Table 3:** Equilibrium water content of BC/PVA crosslinked composite hydrogels. Data are presented as median (Q1–Q3), *n* = 7. Statistical analysis was performed using the Kruskal–Wallis test and effect size (ε^2^).

	BCPVA6	BCPVA9	BCPVA12	BCPVA15	*p*
**I**	91.56 (IQR: 90.96–92.31)	91.57 (IQR: 90.33–91.75)	91.23 (IQR: 90.60–91.60)	91.25 (IQR: 90.81–91.99)	0.767
**II**	89.72 (IQR: 89.20–89.91)	89.63 (IQR: 88.50–90.20)	89.21 (IQR: 88.57–90.74)	91.08 (IQR: 89.55–91.98)	0.175
**III**	88.13 (IQR: 86.66–89.29)	90.30 (IQR: 87.96–90.59)	91.98 (IQR: 90.93–93.35)	94.67 (IQR: 94.11–95.08)	<0.001 ^†^
***p***	0.001 ^‡^	0.055	0.009 ^‡^	0.001 ^‡^	
ε^2^	0.661	0.212	0.417	0.639	

Note: Data are presented as median (Q1–Q3), *n* = 7. ^†^ *p* < 0.05 indicates statistically significant differences among the BCPVA6-III, BCPVA9-III, BCPVA12-III, and BCPVA15-III groups. ^‡^ *p* < 0.05 denotes statistically significant differences within the BCPVA6, BCPVA12, and BCPVA15 groups. ε^2^ represents the effect size derived from the Kruskal–Wallis test. I, II, and III indicate the BC:PVA ratio of 3:1, 1:1, and 1:3, respectively. The symbol *p* represents the statistical *p*-value.

**Table 4:** Pairwise comparisons of the equilibrium water content in BC/PVA crosslinked composite hydrogels using Dunn’s post hoc test with Bonferroni adjustment.

Comparison	*Z* Value	Adjusted *p*-Value	Significance
BCPVA6-I vs. BCPVA6-II	2.800	0.015	*
BCPVA6-I vs. BCPVA6-III	3.532	0.001	*
BCPVA6-II vs. BCPVA6-III	0.732	1.000	ns
BCPVA12-I vs. BCPVA12-II	2.197	0.084	ns
BCPVA12-I vs. BCPVA12-III	−0.776	1.000	ns
BCPVA12-II vs. BCPVA12-III	−2.973	0.009	*
BCPVA15-I vs. BCPVA15-II	0.388	1.000	ns
BCPVA15-I vs. BCPVA15-III	−2.972	0.009	*
BCPVA15-II vs. BCPVA15-III	−3.360	0.002	*
BCPVA6-III vs. BCPVA9-III	−0.877	1.000	ns
BCPVA6-III vs. BCPVA12-III	−2.762	0.034	*
BCPVA6-III vs. BCPVA15-III	−4.3454	0.000	*
BCPVA9-III vs. BCPVA12-III	−1.885	0.357	ns
BCPVA9-III vs. BCPVA15-III	−3.477	0.003	*
BCPVA12-III vs. BCPVA15-III	−1.592	0.668	ns

Note: Pairwise comparisons were performed using Dunn’s post hoc test with Bonferroni correction following the Kruskal–Wallis test. *Z* values and adjusted *p*-values are reported. Statistically significant differences are indicated by an asterisk (*) for *p* < 0.05; ns, denotes non-significance.

**Table 5:** Swelling ratios of BC/PVA crosslinked composite hydrogels. Data are presented as median (Q1–Q3), *n* = 7. Statistical analysis was performed using the Kruskal–Wallis test and effect size (ε^2^).

	BCPVA6	BCPVA9	BCPVA12	BCPVA15	*p*
**I**	451.40 (IQR: 397.43–490.76)	442.51 (IQR: 380.94–460.88)	437.93 (IQR: 415.30–478.41)	487.81 (IQR: 433.42–585.26)	0.144
**II**	664.70 (IQR: 602.19–685.26)	669.05 (IQR: 620.37–800.78)	617.78 (IQR: 548.90–646.09)	670.40 (IQR: 660.51–767.38)	0.119
**III**	554.39 (IQR: 484.27–653.92)	643.30 (IQR: 561.94–679.83)	799.18 (IQR: 604.89–978.01)	997.22 (IQR: 846.9–1029.36)	0.005 ^†^
***p***	0.017 ^‡^	0.001 ^‡^	0.001 ^‡^	<0.001 ^‡^	
ε^2^	0.343	0.661	0.649	0.840	

Note: Data are presented as median (Q1–Q3), *n* = 7. Statistical differences was evaluated using the Kruskal–Wallis test. ^†^ *p* < 0.05 indicates significant differences among the BCPVA6-III, BCPVA9-III, BCPVA12-III, and BCPVA15-III groups. ^‡^ *p* < 0.05 denotes significant differences within the BCPVA6, BCPVA9, BCPVA12, and BCPVA15 group. ε^2^ represents the effect size derived from the Kruskal–Wallis test. I, II, and III indicate the BC:PVA ratio of 3:1, 1:1, and 1:3, respectively. The symbol *p* represents the statistical *p*-value.

**Table 6:** Pairwise comparisons of swelling ratios in BC/PVA crosslinked composite hydrogels using Dunn’s post hoc test with Bonferroni adjustment.

Comparison	*Z* Value	Adjusted *p*-Value	Significance
BCPVA6-I vs. BCPVA6-II	−2.843	0.013	*
BCPVA6-I vs. BCPVA6-III	−1.680	0.279	ns
BCPVA6-II vs. BCPVA6-III	1.163	0.735	ns
BCPVA9-I vs. BCPVA9-II	−3.532	0.001	*
BCPVA9-I vs. BCPVA9-III	−2.800	0.015	*
BCPVA9-II vs. BCPVA9-III	0.732	1.000	ns
BCPVA12-I vs. BCPVA12-II	−2.283	0.067	ns
BCPVA12-I vs. BCPVA12-III	−3.661	0.001	*
BCPVA12-II vs. BCPVA12-III	−1.378	0.504	ns
BCPVA15-I vs. BCPVA15-II	−2.197	0.084	ns
BCPVA15-I vs. BCPVA15-III	−4.135	0.000	*
BCPVA15-II vs. BCPVA15-III	−1.938	0.158	ns
BCPVA6-III vs. BCPVA9-III	−0.390	1.000	ns
BCPVA6-III vs. BCPVA12-III	−1.754	0.476	ns
BCPVA6-III vs. BCPVA15-III	−3.249	0.007	*
BCPVA9-III vs. BCPVA12-III	−1.365	1.000	ns
BCPVA9-III vs. BCPVA15-III	−2.859	0.025	*
BCPVA12-III vs. BCPVA15-III	−1.495	0.810	ns

Note: Pairwise comparisons were performed using Dunn’s post-hoc test with Bonferroni correction following the Kruskal–Wallis test. *Z* values and adjusted *p*-values are reported. Statistically significant differences are indicated by an asterisk (*) for *p* < 0.05; ns, denotes non-significance.

**Table 7:** Compressive strength at 30% strain of BC/PVA crosslinked composite hydrogels. Data are presented as median value (Q1–Q3), *n* = 4. Statistical analysis was performed using the Kruskal–Wallis test and effect size (ε^2^).

	BCPVA6	BCPVA9	BCPVA12	BCPVA15	*p*
**I**	83.85 (IQR: 65.70–89.24)	88.75 (IQR: 87.77–90.22)	62.28 (IQR: 54.43–69.14)	62.76 (IQR: 58.35–66.20)	0.050
**II**	89.73 (IQR: 70.61–93.65)	93.16 (IQR: 91.69–96.11)	90.71 (IQR: 83.85–92.67)	92.18 (IQR: 69.63–95.12)	0.392
**III**	93.16 (IQR: 91.10–95.12)	78.46 (IQR: 67.67–95.62)	90.22 (IQR: 89.79–91.69)	89.24 (IQR: 81.89–93.65)	0.407
***p***	0.118	0.145	0.024 ^†^	0.124	
ε^2^	0.126	0.103	0.301	0.121	

Note: Data are presented as median value (Q1–Q3), *n* = 4. Statistical difference was evaluated using the Kruskal–Wallis test. ^†^ *p* < 0.05 indicates statistically significant differences within the BCPVA12-III groups. ε^2^ represents the effect size derived from the Kruskal–Wallis test. I, II, and III indicate the BC:PVA ratio of 3:1, 1:1, and 1:3, respectively. The symbol *p* represents the statistical *p*-value.

**Table 8:** Dunn’s post hoc test with Bonferroni adjustment for pairwise comparisons of compressive strength among the BCPVA12 groups.

Comparison	*Z* Value	Adjusted *p*-Value	Significance
BCPVA12-I vs. BCPVA12-II	−2.407	0.048	*
BCPVA12-I vs. BCPVA12- III	−2.308	0.063	ns
BCPVA12-II vs. BCPVA12-III	0.098	1.000	ns

Note: Pairwise comparisons were performed using Dunn’s post-hoc test with Bonferroni correction following the Kruskal–Wallis test. *Z* values and adjusted *p*-values are reported. Statistically significant differences are indicated by an asterisk (*) for *p* < 0.05; ns, denotes non-significance.

**Table 9:** The ratio between 1% *w*/*w* BC and 10% *w*/*w* PVA solution by weight.

Group	ZnCl_2_ *n*H_2_O	BC:PVA Ratio	BC Content (wt%)	PVA Content (wt%)
BCPVA6-I	*n* = 6	3:1	0.75	0.25
BCPVA6-II	*n* = 6	1:1	0.50	0.50
BCPVA6-III	*n* = 6	1:3	0.25	0.75
BCPVA9-I	*n* = 9	3:1	0.75	0.25
BCPVA9-II	*n* = 9	1:1	0.50	0.50
BCPVA9-III	*n* = 9	1:3	0.25	0.75
BCPVA12-I	*n* = 12	3:1	0.75	0.25
BCPVA12-II	*n* = 12	1:1	0.50	0.50
BCPVA12-III	*n* = 12	1:3	0.25	0.75
BCPVA15-I	*n* = 15	3:1	0.75	0.25
BCPVA15-II	*n* = 15	1:1	0.50	0.50
BCPVA15-III	*n* = 15	1:3	0.25	0.25

## Data Availability

The data presented in this study are available on request from the corresponding author.
